# A dissemination and education programme to improve the clinical behaviours of psychiatrists in accordance with treatment guidelines for schizophrenia and major depressive disorders: the Effectiveness of Guidelines for Dissemination and Education in Psychiatric Treatment (EGUIDE) project

**DOI:** 10.1192/bjo.2022.44

**Published:** 2022-04-21

**Authors:** Hisashi Yamada, Mikuni Motoyama, Naomi Hasegawa, Kenichiro Miura, Junya Matsumoto, Kazutaka Ohi, Norio Yasui-Furukori, Shusuke Numata, Masahiro Takeshima, Nobuhiro Sugiyama, Tatsuya Nagasawa, Chika Kubota, Kiyokazu Atake, Takashi Tsuboi, Kayo Ichihashi, Naoki Hashimoto, Takahiko Inagaki, Yoshikazu Takaesu, Jun-ichi Iga, Hikaru Hori, Toshiaki Onitsuka, Hiroshi Komatsu, Akitoyo Hishimoto, Kentaro Fukumoto, Michiko Fujimoto, Toshinori Nakamura, Kiyotaka Nemoto, Ryuji Furihata, Satoshi Yamamura, Hirotaka Yamagata, Kazuyoshi Ogasawara, Eiichi Katsumoto, Atsunobu Murata, Hitoshi Iida, Shinichiro Ochi, Manabu Makinodan, Mikio Kido, Taishiro Kishimoto, Yuka Yasuda, Masahide Usami, Taro Suwa, Ken Inada, Koichiro Watanabe, Ryota Hashimoto

**Affiliations:** Department of Neuropsychiatry, Hyogo College of Medicine, Japan; and Department of Pathology of Mental Diseases, National Institute of Mental Health, National Center of Neurology and Psychiatry, Japan; Department of Neuropsychiatry, Hyogo College of Medicine, Japan; Department of Pathology of Mental Diseases, National Institute of Mental Health, National Center of Neurology and Psychiatry, Japan; Department of Pathology of Mental Diseases, National Institute of Mental Health, National Center of Neurology and Psychiatry, Japan; Department of Pathology of Mental Diseases, National Institute of Mental Health, National Center of Neurology and Psychiatry, Japan; Department of Psychiatry, Gifu University Graduate School of Medicine, Japan; Department of Psychiatry, Dokkyo Medical University School of Medicine, Japan; Department of Psychiatry, Graduate School of Biomedical Science, Tokushima University, Japan; Department of Neuropsychiatry Section of Neuro and Locomotor Science, Akita University Graduate School of Medicine, Japan; Department of Psychiatry, Shinshu University School of Medicine, Japan; and Department of Applied Occupational Therapy, Shinshu University School of Health Sciences, Japan; Department of Neuropsychiatry, Kanazawa Medical University, Japan; Department of Pathology of Mental Diseases, National Institute of Mental Health, National Center of Neurology and Psychiatry, Japan; Kyushu Health Administration Center, Nippon Telegraph and Telephone West Corporation, Japan; Department of Neuropsychiatry, Kyorin University School of Medicine, Japan; Department of Neuropsychiatry, University of Tokyo Hospital, Japan; Department of Psychiatry, Hokkaido University Graduate School of Medicine, Japan; Adolescent Mental Health Service, Biwako Hospital, Japan; and Department of Psychiatry, Shiga University of Medical Science, Japan; Department of Neuropsychiatry, Graduate School of Medicine, University of the Ryukyus, Japan; Department of Neuropsychiatry, Molecules and Function, Ehime University Graduate School of Medicine, Japan; Department of Psychiatry, Faculty of Medicine, Fukuoka University, Japan; Department of Neuroimaging Psychiatry, Graduate School of Medical Sciences, Kyushu University, Japan; Department of Psychiatry, Tohoku University Hospital, Japan; Department of Psychiatry, Yokohama City University Graduate School of Medicine, Japan; Department of Neuropsychiatry, Iwate Medical University School of Medicine, Japan; Department of Pathology of Mental Diseases, National Institute of Mental Health, National Center of Neurology and Psychiatry, Japan; and Department of Psychiatry, Osaka University Graduate School of Medicine, Japan; Department of Psychiatry, Shinshu University School of Medicine, Japan; Department of Psychiatry, Faculty of Medicine, University of Tsukuba, Japan; Kyoto University Health Service, Japan; Suzuka Kosei Hospital, Japan; Division of Neuropsychiatry, Department of Neuroscience, Yamaguchi University School of Medicine, Japan; Center for Postgraduate Clinical Training and Career Development, Nagoya University Hospital, Japan; Katsumoto Mental Clinic, Japan; Department of Pathology of Mental Diseases, National Institute of Mental Health, National Center of Neurology and Psychiatry, Japan; Department of Psychiatry, Faculty of Medicine, Fukuoka University, Japan; Department of Neuropsychiatry, Molecules and Function, Ehime University Graduate School of Medicine, Japan; Department of Psychiatry, Faculty of Medicine, Nara Medical University, Japan; Department of Psychiatry, Toyama City Hospital, Japan; and Department of Neuropsychiatry, University of Toyama Graduate School of Medicine and Pharmaceutical Sciences, Japan; Department of Neuropsychiatry, Keio University School of Medicine, Japan; Department of Pathology of Mental Diseases, National Institute of Mental Health, National Center of Neurology and Psychiatry, Japan; and Life Grow Brilliant Mental Clinic, Medical Corporation Foster, Japan; Department of Child and Adolescent Psychiatry, Kohnodai Hospital, National Center for Global Health and Medicine, Japan; Department of Psychiatry, Graduate School of Medicine, Kyoto University, Japan; Department of Psychiatry, Tokyo Women's Medical University, Japan; Department of Neuropsychiatry, Kyorin University School of Medicine, Japan; Department of Pathology of Mental Diseases, National Institute of Mental Health, National Center of Neurology and Psychiatry, Japan

**Keywords:** Clinical practice guidelines, educational programme, schizophrenia, major depressive disorder, EGUIDE project

## Abstract

**Background:**

Clinical practice guidelines for schizophrenia and major depressive disorder have been published. However, these have not had sufficient penetration in clinical settings. We developed the Effectiveness of Guidelines for Dissemination and Education in Psychiatric Treatment (EGUIDE) project as a dissemination and education programme for psychiatrists.

**Aims:**

The aim of this study is to assess the effectiveness of the EGUIDE project on the subjective clinical behaviour of psychiatrists in accordance with clinical practice guidelines before and 1 and 2 years after participation in the programmes.

**Method:**

A total of 607 psychiatrists participated in this study during October 2016 and March 2019. They attended both 1-day educational programmes based on the clinical practice guidelines for schizophrenia and major depressive disorder, and answered web questionnaires about their clinical behaviours before and 1 and 2 years after attending the programmes. We evaluated the changes in clinical behaviours in accordance with the clinical practice guidelines between before and 2 years after the programme.

**Results:**

All of the scores for clinical behaviours in accordance with clinical practice guidelines were significantly improved after 1 and 2 years compared with before attending the programmes. There were no significant changes in any of the scores between 1 and 2 years after attending.

**Conclusions:**

All clinical behaviours in accordance with clinical practice guidelines improved after attending the EGUIDE programme, and were maintained for at least 2 years. The EGUIDE project could contribute to improved guideline-based clinical behaviour among psychiatrists.

## Clinical practice guidlines for psychiatric disorders

Clinical practice guidelines provide recommendations for optimising patient treatment, and are based on a systematic review of evidence and an assessment of the advantages and disadvantages of alternative care options and standard tools for clinical decision-making. Various guidelines for the clinical practice of psychiatric disorders have been published,^1–8^ and in many countries, psychiatrists commonly make clinical decisions based on clinical practice guidelines. In Japan, clinical practice guidelines for psychiatric disorders were only published 9 years ago, and Japanese psychiatrists usually make clinical decisions based on their own experience or knowledge and not based on clinical practice guidelines. As a result, pharmacotherapy for psychiatric disorders in Japan has been different from that recommended in clinical practice guidelines in other countries.^9–14^ To change this situation, the Japanese Society of Neuropsychopharmacology published the ‘Guideline for Pharmacological Therapy of Schizophrenia’ (clinical practice guideline for schizophrenia) in 2015,^15^ and the Japanese Society of Mood Disorders published the ‘Treatment Guideline: Major Depressive Disorder’ in 2012,^16^ which was revised to the ‘Treatment Guideline II: Major Depressive Disorder’ (clinical practice guideline for major depressive disorder) in 2016.^17^

## Dissemination of clinical practice guidelines in Japan

Although the clinical practice guidelines of schizophrenia and major depressive disorder have been published, pharmacotherapy for these disorders has not undergone sufficient transformation in Japan.^9,10^ For example, our project previously showed that for patients with schizophrenia, 57.1% were prescribed antipsychotic monotherapy, 15.5% were prescribed antipsychotic monotherapy without any other psychotropics and 31.7% received no prescription of anxiolytics or hypnotics. In addition, at 84 institutions before doctors participated in the educational programmes, 58.6% of patients with depression undergoing in-patient treatment were prescribed antidepressant monotherapy and 25.1% received no prescription of anxiolytics or hypnotics.^18,19^ To improve these statistics, dissemination of and education on the guidelines for Japanese psychiatrists was needed. Thus, we launched the Effectiveness of Guidelines for Dissemination and Education in Psychiatric Treatment (EGUIDE) project in 2016. The purpose of the EGUIDE project is to disseminate the guidelines by conducting educational programmes on the clinical practice guidelines for schizophrenia and major depressive disorder for psychiatrists, and to standardise medical practices in accordance with the clinical practice guidelines. We have already reported on the educational method and the effectiveness of the EGUIDE project in improving knowledge of the clinical practice guidelines in psychiatrists.^20^ Additionally, the effectiveness of the educational programmes was investigated by evaluating psychiatrists’ clinical behaviours in accordance with the clinical practice guidelines.

The aim of this research is to assess the efficacy of the EGUIDE project in changing clinical behaviours in accordance with the clinical practice guidelines in psychiatrists before and 1 and 2 years after they attend the programmes.

## Method

### Design and participants

The EGUIDE project recruited psychiatrists from >100 medical institutions in Japan, who volunteered to participate in study during October 2016 and March 2019. All participants signed informed written consent forms. This study was approved by the ethics committee at the National Center of Neurology and Psychiatry (approval number A2017-105) and each of the participating universities, hospitals and clinics. The procedures were carried out in accordance with the Helsinki Declaration. The study protocol was registered in the University Hospital Medical Information Network registry (identifier UMIN000022645). The participants attended a 1-day educational programme on schizophrenia and depression based on the clinical practice guideline for schizophrenia (published by the Japanese Society of Neuropsychopharmacology) and the clinical practice guideline for major depressive disorder (published by the Japanese Society of Mood Disorders). We conducted lectures on the guidelines and discussions using two clinical cases to describe the guidelines and how to apply them in practice. The participants received emails including URLs for self-administered web questionnaires. Using the questionnaires, they retrospectively rated their clinical behaviours in accordance with the clinical practice guidelines in the 6 months before the programme they attended, and they again rated themselves each year thereafter, for 2 years. The effectiveness of each programme was assessed based on changes in the scores of the self-administered questionnaires before and after the programmes.

### Assessment measures

To assess participants’ clinical behaviours with respect to general use of the clinical guidelines, a self-administered questionnaire was created consisting of six items rated on a five-point Likert scale (Supplementary Table 1 available at https://doi.org/10.1192/bjo.2022.44). Participants assessed their clinical behaviours in accordance with the clinical practice guidelines for schizophrenia and major depressive disorder by creating self-administered questionnaires, each consisting of 14 items rated on a five-point Likert scale (Supplementary Tables 2 and 3). The evaluations of clinical behaviours were divided into the following six stages, according to the degree of achievement: not achieved (0–20% achieved), slightly achieved (21–40% achieved), approximately half achieved (41–60% achieved), moderately achieved (61–80% achieved), almost achieved (81–100% achieved) and no opportunity.

### Statistical analysis

As the representative value for each of the five achievement levels, an intermediate value was used: 10 for ‘not achieved’, 30 for ‘slightly achieved’, 50 for ‘about half achieved’, 70 for ‘moderately achieved’ and 90 for ‘almost achieved’. Therefore, there were five scores, and the scores ranged from 10 to 90. We excluded ‘no opportunity’ from the analysis. We used the Kolmogorov–Smirnov test to evaluate data normality in the scores for the clinical behaviours. We assessed the homoscedasticity of variance with Levine's test. Data were analysed by Kruskal–Wallis test with statistical significance, because the Kolmogorov–Smirnov test did not show normal distribution or homoscedasticity. To compare the changes in the scores for the clinical behaviours between before and 1 and 2 years after attending the programme, the Bonferroni correction was applied for multiple testing when the Kruskal–Wallis test was statistically significant. A significance threshold of 0.05 was applied for multiple testing. The data were initially imputed in Microsoft Excel for Mac (Microsoft Corp, 2010), and then analysed with SPSS version 24.0 for Mac (IBM SPSS Inc, 2016).

## Results

### Demographics of the participants

A total of 607 psychiatrists from 134 medical institutions participated in educational programmes on the clinical guidelines for treatment of schizophrenia and depression during the 3 years of the EGUIDE project recruitment (October 2016 to March 2019). All of the participants attended a 1-day educational programme on schizophrenia and depression based on the clinical practice guidelines. Of these, 594 participants joined the email-based survey, 425 responded to the web questionnaires at baseline, 270 responded at 1 year and 140 responded at 2 years. Some participants have dropped out during each follow-up period. The numbers of participants who answered web questionnaires, excluding those who dropped out and those who reported ‘no opportunity’ for general use of the clinical guidelines at baseline and after the programmes, are shown in Supplementary Tables 4–6. They were aged 26–70 years, with an average age of 34.4 ± 7.6 years; 369 (72.5%) were male. Years of professional experience ranged from 1 to 35 years, with an average of 5.7 ± 6.7 years. A total of 463 belonged to university hospitals, 20 belonged to psychiatric hospitals, 25 belonged to general hospitals and one belonged to a clinic when attending the programme.

### Changes in the scores for clinical behaviours in accordance with the pre- and post-programme

**Fig. 1:**
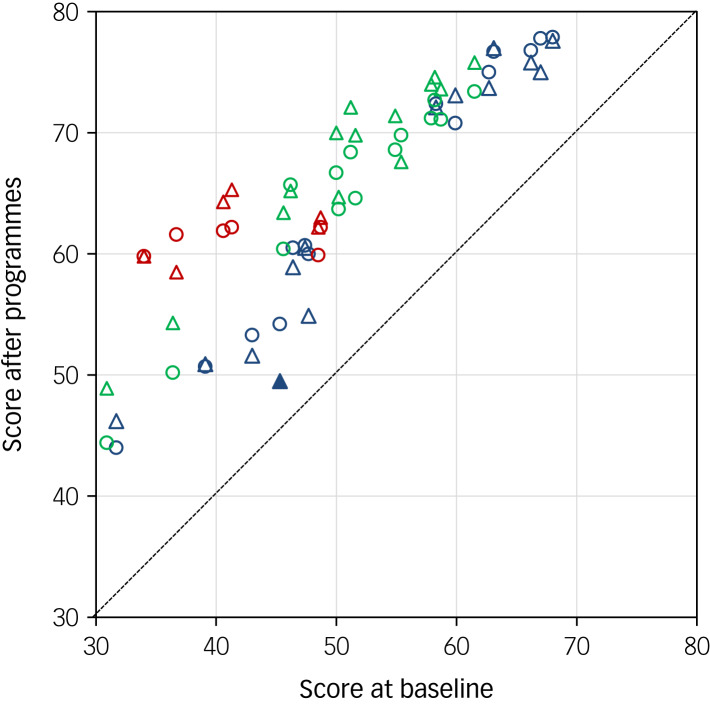
Comparison of clinical behaviour scores for the use of clinical guidelines at baseline and after the ‘Guideline for Pharmacological Therapy of Schizophrenia’ and ‘Treatment Guideline II: Major Depressive Disorder’ programmes. The *x*- and *y*-axes indicate the score for each question at baseline and the score for each question after programme participation, respectively. Details of each score are shown in Tables 1–3. Blue circles indicate clinical behaviour scores that increased significantly 1 year after attending the ‘Guideline for Pharmacological Therapy of Schizophrenia’ programme, compared with before the course (S1–S14). Blue triangles indicate clinical behaviour scores that increased significantly 2 years after attending the ‘Guideline for Pharmacological Therapy of Schizophrenia’ programme, compared with before the course (S1–S7, S9–S14). Green circles indicate clinical behaviour scores that increased significantly 1 year after attending the ‘Treatment Guideline II: Major Depressive Disorder’ programme, compared with before the course (D1–D14). Green triangles indicate clinical behaviour scores that increased significantly 2 years after attending the ‘Treatment Guideline II: Major Depressive Disorder’ programme, compared with before the course (D1–D14). Red circles indicate clinical behaviour scores that increased significantly 1 year after attending the ‘Guideline for Pharmacological Therapy of Schizophrenia’ and ‘Treatment Guideline II: Major Depressive Disorder’ programmes, compared with before the course (G1–G6). Red triangles indicate clinical behaviour scores that increased significantly 2 years after attending the ‘Guideline for Pharmacological Therapy of Schizophrenia’ and ‘Treatment Guideline II: Major Depressive Disorder’ programmes, compared with before the course (G1–G6). Solid blue triangles indicate clinical behaviour scores that were not significantly elevated 2 years after attending the ‘Guidelines for the Pharmacotherapy of Schizophrenia’ programme compared with before the programme (S8).

A comparison of all clinical behaviour scores in accordance with the guidelines at baseline and after the clinical practice guideline programmes is shown in Fig. 1. All of the mean scores had increased significantly at 1 year after attending the programme. Furthermore, the clinical behaviours of participants were still significantly higher at 2 years compared with the baseline, with the exception of ‘Choosing treatment with modified electroconvulsive therapy for patients with treatment-resistant schizophrenia’.

The mean and statistical results of the clinical behaviours in accordance with the general use of the guidelines for before and 2 years after programme participation are shown in Table 1, and the data of multiple comparisons are shown in Supplementary Table 7. In all subclasses of clinical behaviours consistent with the general use of the clinical guidelines, the mean scores increased significantly after attending the programme. Furthermore, the clinical behaviours of participants were maintained for 2 years, as the mean score was not significantly different between 1 and 2 years after the programme. One year after attending the programme, a large and significant change was observed in ‘Using treatment guidelines when deciding on the treatment policy in discussions with patients and family’ (*Z* = 12.75, *P* = 3.2 × 10^−37^, *r* = 0.50). Additionally, moderate and significant changes were seen in ‘Trying to treat patients in accordance with guidelines if their previous treatments are not in accordance with guidelines’ (*Z* = 12.81, *P* = 1.4 × 10^−37^, *r* = 0.50), ‘Recommending pharmacotherapy for schizophrenia to fellow doctors in accordance with the guideline’ (*Z* = 9.94, *P* = 2.9 × 10^−23^, *r* = 0.39) and ‘Recommending treatment for depression to fellow doctors in accordance with the guideline’ (*Z* = 9.83, *P* = 8.7 × 10^−23^, *r* = 0.39).

**Table 1:** Comparison of clinical behaviour scores for the general use of clinical guidelines at baseline and after the programmes

		Baseline		One year later		Two years later		Statistics^a^	
		Mean	s.d.	Mean	s.d.	Mean	s.d.	H	*P*-value
G1	Using treatment guidelines when deciding on the treatment policy in discussions with patients and family	34.0	±22.2	59.8^b^	±21.3	59.8^c^	±21.0	204.97	3.1 × 10^−45^
G2	Trying to treat patients in accordance with the guidelines if their previous treatments are not in accordance with guidelines	36.7	±21.8	61.6^b^	±20.0	58.5^c^	±21.1	192.27	1.8 × 10^−42^
G3	Pharmacotherapy for schizophrenia in your hospital/clinic is in accordance with the guideline	48.5	±22.4	59.9^b^	±21.7	62.2^c^	±21.4	62.40	2.8 × 10^−14^
G4	Recommending pharmacotherapy for schizophrenia to fellow doctors in accordance with the guideline	40.6	±24.5	61.9^b^	±23.2	64.3_c_	±21.4	138.20	9.8 × 10^−31^
G5	Treatment for depression in your hospital/clinic is in accordance with the guideline	48.7	±23.4	62.2^b^	±20.0	63.0^c^	±21.5	73.49	1.1 × 10^−16^
G6	Recommending treatment for depression to fellow doctors in accordance with the guideline	41.3	±24.3	62.2^b^	±23.5	65.3^c^	±20.8	138.38	8.9 × 10^−31^

The complete questions are noted in Supplementary Table 1. An intermediate value was used as the representative value for each of the five achievement levels: 0–20, 21–40, 41–60, 61–80 and 81–100. The scores ranged from 10 to 90.

a. The Kruskal–Wallis test was used for the statistical analysis as the Kolmogorov–Smirnov test did not indicate normal distribution of clinical behaviour scores at baseline or 1 or 2 years after the programme. The significance level was set at <0.05.

b. The mean scores of clinical behaviours increased significantly 1 year after attending the programme compared with baseline.

c. The mean scores of clinical behaviours increased significantly 2 years after attending the programme compared with baseline.

Table 2 shows the changes in clinical behaviour scores before and after attending the educational programme for the clinical practice guideline for schizophrenia. Supplementary Table 8 shows the multiple comparison of clinical behaviour scores at baseline and 1 and 2 years after the programmes. The mean scores of all of the subclasses increased significantly after the programme. The clinical behaviours of participants, except ‘Choosing treatment with modified electroconvulsive therapy for patients with treatment-resistant schizophrenia’, were maintained for 2 years. A moderate and significant change was observed in ‘Choosing antipsychotic monotherapy but not a combination of antipsychotics’ (*Z* = 8.04, *P* = 9.0 × 10^−16^, *r* = 0.31) 1 year after attending the programme.

**Table 2:** Comparison of clinical behaviour scores at baseline and after the ‘Guideline for Pharmacological Therapy of Schizophrenia’ programme

		Baseline		One year later		Two years later		Statistics^a^	
		Mean	s.d.	Mean	s.d.	Mean	s.d.	H	*P*-value
S1	Choosing antipsychotic monotherapy but not a combination of antipsychotics	46.4	±22.9	60.5^b^	±19.4	58.9^c^	±20.3	75.74	3.6 × 10^−17^
S2	Refraining from using psychotropic drugs other than antipsychotics	31.7	±20.9	44.0^b^	±23.8	46.2^c^	±21.4	68.10	1.6 × 10^−15^
S3	Providing continuous guidance on the daily administration of antipsychotics	66.2	±23.7	76.8^b^	±17.4	75.8^c^	±18.9	40.39	1.7 × 10^−9^
S4	Ensuring the appropriate dose and timing of pharmacological treatment and the extent of medication adherence in treatment for recurrence or relapse of schizophrenia	62.7	±23.5	75.0^b^	±18.1	73.7^c^	±20.3	56.86	4.5 × 10^−13^
S5	Choosing medication considering the response to medications in the past in treatment for recurrence or relapse of schizophrenia	63.1	±23.3	76.7^b^	±17.1	77.0^c^	±17.4	78.02	1.1 × 10^−17^
S6	Defining those with treatment-resistant schizophrenia as patients with schizophrenia who, despite taking at least two antipsychotics with adequate doses and timing, have persistent symptoms	58.3	±26.9	72.4^b^	±20.7	72.1^c^	±21.4	54.31	1.6 × 10^−12^
S7	Choosing treatment with clozapine for patients with treatment-resistant schizophrenia	43.0	±27.5	53.3^b^	±27.9	51.6^c^	±26.4	15.75	3.8 × 10^−4^
S8	Choosing treatment with modified electroconvulsive therapy for patients with treatment-resistant schizophrenia	45.3	±25.9	54.2^b^	±26.1	49.5	±27.5	12.35	2.1 × 10^−3^
S9	Continuing administration of antipsychotics for at least 1 year for first episode psychosis for relapse prevention	67.0	±23.8	77.8^b^	±17.6	75.0^c^	±19.5	36.14	1.4 × 10^−8^
S10	Choosing long-acting injection antipsychotics for patients whose relapse is due to low medication adherence	47.7	±23.8	60.0^b^	±24.1	54.9_c_	±25.6	36.13	1.4 × 10^−8^
S11	For recovery from cognitive impairment in schizophrenia, refraining from using anticholinergics	47.4	±24.5	60.7^b^	±22.9	60.5^c^	±21.2	57.31	3.6 × 10^−13^
S12	For recovering from cognitive impairment in schizophrenia, refraining from using benzodiazepines	39.1	±23.3	50.7_b_	±24.3	50.9^c^	±24.1	46.84	6.7 × 10^−11^
S13	Choosing second-generation antipsychotics to decrease the possibility of extrapyramidal adverse effects	68.0	±21.9	77.9^b^	±16.5	77.6^c^	±16.9	46.33	8.7 × 10^−11^
S14	Choosing oral medication for the management of psychomotor agitation if possible	59.9	±23.1	70.8_b_	±20.0	73.1^c^	±19.4	55.92	7.2 × 10^−13^

The complete questions are noted in Supplementary Table 2. An intermediate value was used as the representative value for each of the five achievement levels: 0–20, 21–40, 41–60, 61–80 and 81–100. The scores ranged from 10 to 90.

a. The Kruskal–Wallis test was used for the statistical analysis as the Kolmogorov–Smirnov test did not indicate normal distribution of clinical behaviour scores at baseline or 1 or 2 years after the programme. The significance level was set at <0.05.

b. The mean scores of clinical behaviours increased significantly 1 year after attending the programme compared with baseline.

c. The mean scores of clinical behaviours increased significantly 2 years after attending the programme compared with baseline.

The following are notable points about several general clinical behaviours whose results differed from those previously reported in other countries. The mean score of ‘Choosing antipsychotic monotherapy but not a combination of antipsychotics’ increased after the programme, and a significant change was observed (H = 75.74, *P* = 3.5 × 10^−17^): the score was approximately 60 after the programme (46.4 at baseline, 60.5 at 1 year after the programme and 58.9 at 2 years after the programme). In addition, the mean score for ‘Refraining from using psychotropic drugs other than antipsychotics’ also increased significantly after the programme (H = 68.10, *P* = 1.6 × 10^−15^), but the score was still <50 after the programme (31.7 at baseline, 44.1 at 1 year after the programme and 46.2 at 2 years after the programme). Although the score increased throughout the programme for ‘For recovery from cognitive impairment in schizophrenia, refraining from using benzodiazepines’ (H = 46.84, *P* = 6.7 × 10^−11^), the score at 2 years after the programme was 50.9.

Table 3 shows the mean values and the statistical results of the clinical behaviours before and after the programme for the clinical practice guideline for major depressive disorder. The multiple comparisons of the clinical behaviour scores at baseline and 1 and 2 years after the programme are shown in Supplementary Table 9. In all subclasses, the mean scores increased significantly after attending the programme, and the clinical behaviours were maintained for 2 years. One year after attending programme, moderate changes were observed in ‘Diagnosing depression, including the classification of the severity, based on the DSM-5’ (*Z* = 9.66, *P* = 4.6 × 10^−22^, *r* = 0.37), ‘In diagnosis, assessing information from any person other than the patient and functional impairments before the onset’ (*Z* = 8.27, *P* = 1.4 × 10^−16^, *r* = 0.32), ‘When the treatment does not work well, reassessing the diagnosis, pharmacotherapy and environmental management’ (*Z* = 8.65, *P* = 5.2 × 10^−18^, *r* = 0.33), ‘For mild depression, adding cognitive–behavioural therapy and new-generation antidepressants to fundamental intervention if necessary’ (*Z* = 9.23, *P* = 2.7 × 10^−10^, *r* = 0.36) and ‘For sleep disorders, providing sleep hygiene instructions before pharmacotherapy’ (*Z* = 8.72, *P* = 2.7 × 10^−18^, *r* = 0.34).

**Table 3:** Comparison of clinical behaviour scores at baseline and after the ‘Treatment Guideline II: Major Depressive Disorder’ programme

		Baseline		One year later		Two years later		Statistics^a^	
		Mean	s.d.	Mean	s.d.	Mean	s.d.	H	*P*-value
D1	Diagnosing depression, including the classification of the severity, based on the DSM-5	46.2	±25.3	65.7^b^	±22.8	65.2^c^	±21.1	116.36	5.4 × 10^−26^
D2	In diagnosis, assessing information from any person other than the patient and functional impairments before the onset	55.4	±22.6	69.8^b^	±18.8	67.6^c^	±20.2	79.14	6.5 × 10^−18^
D3	Focusing on empathic or supportive care and performing fundamental interventions such as psychological education first	61.5	±22.6	73.4^b^	±18.8	75.8^c^	±18.1	73.62	1.0 × 10^−16^
D4	When the treatment does not work well, reassessing the diagnosis, pharmacotherapy and environmental management	58.2	±22.4	72.7^b^	±18.5	74.6^c^	±16.9	101.99	7.1 × 10^−23^
D5	For mild depression, adding cognitive–behavioural therapy and new-generation antidepressants to fundamental intervention if necessary	50.0	±22.6	66.7^b^	±20.1	70.0^c^	±21.0	120.00	8.7 × 10^−27^
D6	For moderate/severe depression, using antidepressant monotherapy with adequate doses and timing and considering modified electroconvulsive therapy if necessary	58.7	±23.5	71.1^b^	±20.3	73.6^c^	±19.8	69.70	7.3 × 10^−16^
D7	For moderate/severe depression, if antidepressants are effective but not enough, treating with lithium or antipsychotics or T3/T4 as augmentation therapy	51.6	±25.1	64.6^b^	±24.2	69.8^c^	±22.0	67.84	1.9 × 10^−15^
D8	Refraining from using long-term administration of anxiolytics	36.4	±22.0	50.2^b^	±24.4	54.3^c^	±23.0	81.05	2.5 × 10^−18^
D9	Refraining from using long-term administration of hypnotics	30.9	±21.0	44.4^b^	±23.3	48.9^c^	±23.8	87.01	1.3 × 10^−19^
D10	For psychotic depression, using a combination of antidepressants and antipsychotics	57.9	±25.3	71.2^b^	±20.3	74.0^c^	±17.1	62.49	2.7 × 10^−14^
D11	For psychotic depression, using modified electroconvulsive therapy	50.2	±25.5	63.7^b^	±23.8	64.7^c^	±23.5	42.44	6.1 × 10^−10^
D12	For depression in children and adolescents, providing environmental management, psychological education, supportive intervention and family support before pharmacotherapy	54.9	±25.5	68.6^b^	±22.1	71.4^c^	±20.2	52.48	4.0 × 10^−12^
D13	For sleep disorders, considering differential diagnosis of primary sleep disorders such as obstructive sleep apnoea syndrome first	45.6	±25.2	60.4^b^	±24.9	63.4^c^	±23.4	74.39	7.0 × 10^−17^
D14	For sleep disorders, providing sleep hygiene instructions before pharmacotherapy	51.2	±24.8	68.4^b^	±22.6	72.1^c^	±19.7	112.55	3.6 × 10^−25^

The complete questions are noted in Supplementary Table 3. An intermediate value was used as the representative value for each of the five achievement levels: 0–20, 21–40, 41–60, 61–80 and 81–100. The scores ranged from 10 to 90.

a. The Kruskal–Wallis test was used for the statistical analysis as the Kolmogorov–Smirnov test did not indicate normal distribution of the clinical behaviour scores at baseline or 1 or 2 years after the programme. The significance level was set at <0.05.

b. The mean scores of clinical behaviours increased significantly 1 year after attending the programme compared with baseline.

c. The mean scores of clinical behaviours increased significantly 2 years after attending the programme compared with baseline.

The following are notable points about several general clinical behaviours whose results differed from those previously reported in other countries. The score for the clinical behaviour ‘Refraining from using long-term administration of anxiolytics’ increased significantly throughout the programme (H = 81.05, *P* = 2.5 × 10^−18^), but it was as low as approximately 50 after attending the programme (36.4 at baseline, 50.2 at 1 year after the programme and 54.3 at 2 years after the programme). Although a significant change was also observed in ‘Refraining from using long-term administration of hypnotics’ (H = 87.01, *P* = 1.2 × 10^−19^), the score was <50 after the programme (30.9 at baseline, 44.4 at 1 year after the programme and 48.9 at 2 years after the programme).

## Discussion

This is the first study to investigate the effectiveness of the educational programme in the improvement of psychiatrists’ clinical behaviours in accordance with the clinical practice guidelines.

A total of 607 psychiatrists from 134 medical institutions participated in educational programmes on the clinical guidelines for treatment of schizophrenia and depression during the 3 years of the EGUIDE project recruitment, and all clinical behaviours in accordance with clinical practice guidelines improved after attending the EGUIDE programme and were maintained for at least 2 years. These results suggest that our educational programmes could help to improve psychiatrists’ clinical behaviours in accordance with the guidelines. To the best of our knowledge, no study has shown that educational programmes for clinical practice guideline have led to sustained improvements in clinical behaviour over years.

Previous studies have indicated that there could be a large gap between the development of evidence-based guidelines and their implementation in clinical settings,^18,19,21–23^ and that a combination of several guideline dissemination and implementation strategies aimed at healthcare professionals has failed to reduce antipsychotic polypharmacy for schizophrenia out-patients.^24^ The pathway from evidence to guidelines is highly developed, but the development of guideline implementation strategies has been insufficient and examined in only a few studies.^22,25–27^ Although the barriers to improving guideline adherence have not yet been generalised, some reports suggest that low awareness and dissemination of guidelines, as well as inadequate supply systems, could affect their implementation.^22,26^ In this regard, the EGUIDE project has set up a supply system and provides the opportunity to learn about and become proficient in the guidelines.

The number of psychiatrists in Japan is almost 16 000, and approximately 1000 psychiatrists have already participated in the education programmes in the past 5 years. In 2020, only 6.3% of psychiatrists had completed these programmes, but the EGUIDE project estimates that 2000 psychiatrists will finish the programmes in the next 5 years. If >10% of psychiatrists in Japan achieve improvements in clinical behaviour in accordance with the clinical practice guidelines, treatment for schizophrenia and depression could change, which could lead to an improvement in quality of life for patients in Japan.

To better disseminate the clinical practice guidelines, the education programmes of the EGUIDE project need further improvement, and we seek to improve the delivery method annually. We revised the lecture materials associated with items for which knowledge was considered insufficient, and reported results suggesting that the revision of the lecture materials may have improved the degree of understanding of the clinical practice guidelines.^28^

This study has several limitations that should be taken into account when interpreting the results. First, because this study was performed in a single-arm design without a control group, it was difficult to assess the precise effectiveness of the programme despite it being a before-and-after comparison study. Second, since the questionnaires used to evaluate clinical behaviours in accordance with clinical practice guidelines were not validated, and we used a subjective method of assessment, it was unclear whether the questionnaire could adequately assess whether clinical behaviours were in accordance with clinical practice guidelines. Third, because of the lack of background information on the participants, we presumed that there might be many potential confounding factors related to the improvement of clinical behaviours. Fourth, although an annual web questionnaire survey works well as a reminder for past participants to recall the content of the educational programmes and make them aware of whether clinical behaviours are in accordance with clinical practice guidelines, this evaluation is subjective. To assess the precise effect of the education programme, it is necessary to assess changes in quality indicators, such as the prescriptions issued by participants. The results for the improvement of clinical knowledge of the clinical practice guidelines have already been published,^20^ and in-patient prescribing behaviour will be the object of another paper.

Despite these limitations, the educational programme of the EGUIDE project is considered to be an effective means of guideline dissemination and education. Further dissemination of clinical practice guidelines for schizophrenia and major depression in a variety of clinical settings is needed.

In conclusion, the EGUIDE project, as a dissemination and education programme for the clinical practice guidelines for schizophrenia and major depressive disorder, has the potential to contribute to improvement in clinical behaviours of psychiatrists in accordance with the guidelines.

Further research is needed to clarify the effectiveness of the EGUIDE project for the improvement of quality indicators in clinical situations.

## Data Availability

The data are not for public use due to privacy and ethical restrictions (informed consent has not been obtained for the public availability of raw data).

## Supplementary material

For supplementary material accompanying this paper visit http://doi.org/10.1192/bjo.2022.44. click here to view supplementary material
